# A case of conjoined twins (thoraco-omphalopygopagus tribrachius tetrapus) in lamb 

**Published:** 2014

**Authors:** Yazdan Mazaheri, Jamal Nourinezhad, Reza Ranjbar, Mahmood Khaksary Mahabady, Ali Reza Ghadiri, Hamid Lombeshkon

**Affiliations:** 1 *Department of Basic Sciences, Faculty of Veterinary Medicine, Shahid Chamran University, Ahvaz, Iran; *; 2 *Department of Clinical Sciences, Faculty of Veterinary Medicine, Shahid Chamran University, Ahvaz, Iran; *; 3 *Private Veterinary Practitioner, Shushtar, Iran.*

**Keywords:** Conjoined twins, Diplopagus, Notomelus, Sheep, Tribrachius

## Abstract

Nearly completed conjoined or fused symmetrical twins are generally called diplopagus. Sheep conjoined twins have been reported less than cow. An apparently female conjoined twin lambs was examined based on external and internal features. In radiology, two vertebral columns and two pairs of the ribs were seen. Only two heads and two necks were separated (thoraco-omphalopygopagus). There were three forelimbs (tribrachius), one of which grew on dorsal region as a notomelus. Teat buds of the monsters differed in number. Only one lamb had umbilicus, including one umbilical vein, and two umbilical arteries locating besides one urinary bladder. This lamb had also one uterus. Two-separated alimentary tracts were observed in a common abdomen. Common thorax and abdominal cavities were separated by a diaphragm. There were two esophageal hiatuses, and two caval foramina but only one aortic hiatus. Two pairs of lungs and two unequal and connected hearts in a common pericardium were observed. Abnormality of the circulatory system might have caused the death of the twins.

## Introduction

Occasionally, separation of the two portions of the embryo is incomplete resulting in the formation of conjoined twins.^1^^,^^2^ Definitive etiological information and data about embryonic duplications are limited.^3^^,^^4^ Misexpression of gene such as goosecoid may be involved in this malformation. Conjoined twins can be separated only when they have no vital parts in common.^1^

Conjoined twins are considered to be more common in cattle than in other domestic animals.^3^^,^^5^ Anomalous twins are extremely rare in horses while they are not uncommon in pigs and sheep, and occur occasionally in dogs and cats. More recently, Binati and Riccaboni found thoraco-omphalopagus conjoined twins in a goat.^6^ Dennis examined 4,417 lambs for indicating the causes of the prenatal lamb mortalities, which 401 of them were congenitally malformed and 27 cases had embryonic duplications.^3^

Cywes *et al*. studied 25 sets of conjoined twins, containing 14 complete and symmetrical sets and 11 incomplete or heteropagus.^7^ Castilla *et al.* reported sex distribution was even in conjoined human twins.^8^ Recent report indicated the incidence of female conjoined twins is more than male ones.^9^

**Case presentation**

**Fig. 1:**
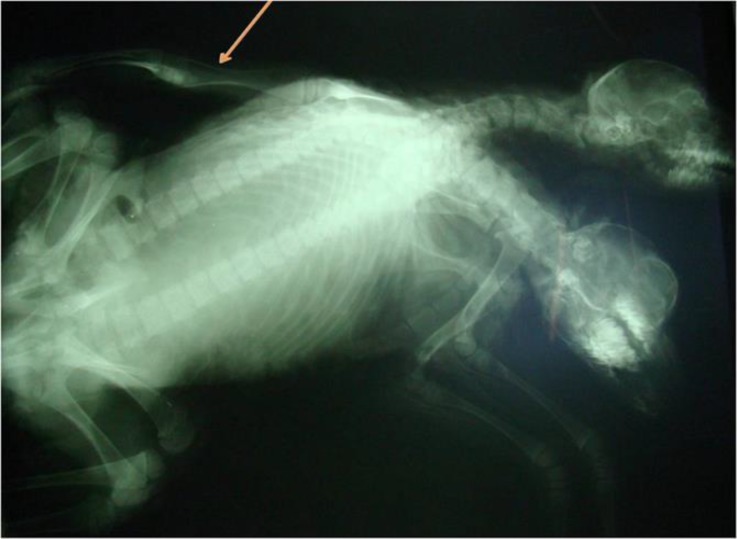
The radiograph shows conjoined twin lambs having two vertebral columns (dispinous). Note the notomelus over N2 (Arrow).

**Fig. 2:**
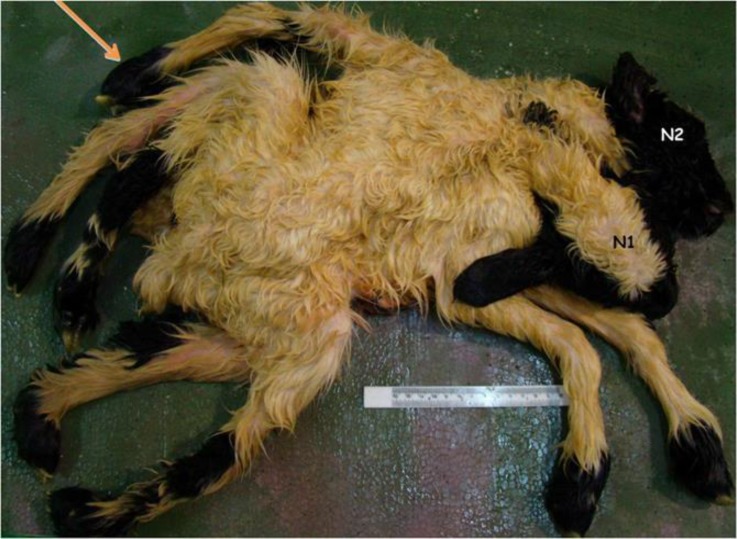
External feature of conjoined twin lambs. Showing the notomelus (Arrow) and in N2 and over extension of the hind limbs in N1

**Fig. 3:**
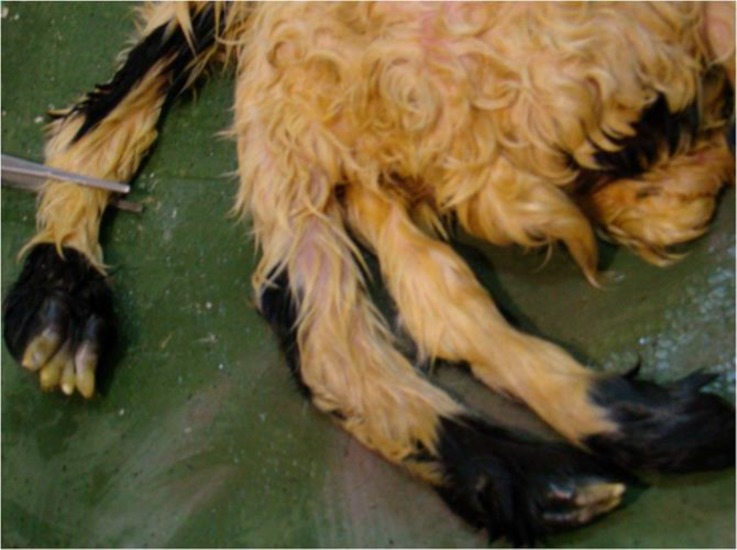
The notomelus shows some digitations in the distal extremity

 A set of apparently female conjoined twin lambs with two separated heads and necks was delivered by cesarean section in Shushtar, southwest of Iran. They were alive for few minutes and then were brought to our department after death. Before external and internal examinations, some radiographs were taken. For easy interpretation, twins were named as N1 (with umbilicus) and N2 (without umbilicus). The whole weight, crown rump length (CRL), limbs length, and the height of mandible at the level of behind the diastema were recorded. Internal examination was performed through umbilical region. Radiographs revealed two vertebral columns (dispinous) and two sets of ribs (Fig. 1). Two heads and necks were separated, but the rest of the two bodies except for the tails were fused (thoraco-omphalopygopagus) (Fig. 2). There were four openings (two ani and two vulvas) locating in a line between two fat tails. The two ani were positioned close to each other (Fig. 3). Total weight of the monster was 5.6 kg. Data of some other dimensions are shown in Table 1. 

**Table 1:** Some dimensions of the new-born apparently female conjoined twin lambs

**Twins**	**CRL**	**R / L**	**FLL (cm)**	**HLL (cm)**	**MH (mm)**
**N1**	38	R	36	36	18
		L	38	36	14
**N2**	39	R	36	36	16
L	notomelus	36	16

CRL: crown- rump length; R: right; L: left; FLL: length of forelimbs; HLL: length of hind limbs; MH: height of the mandible in the level of behind the diastema.

**Fig. 4:**
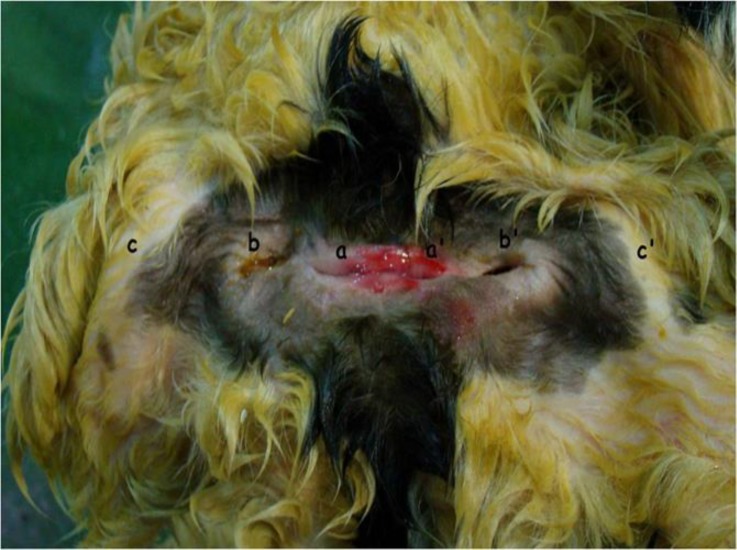
Caudal aspect of the conjoined twin lambs showing two vulvas (a and a'), two anuses (b and b') and two fat tails (c and c').

There were three unequal forelimbs (tribrachius), two of them (belonging to N1) were normal (Fig. 2), and one of them showing on the dorsal region of N2 as a notomelus showing some digitations (Fig. 4). 

There were four apparently normal hind limbs (tetrapus). Those of belonging to N1 showed some over-extension (Fig. 2). There were two teat buds in N1 but five in N2. There was only one umbilical vein and two umbilical arteries. There were two-dental papillae in N1 nothing was observed in N2. Mandibular bone diameters differed for each halves in N1 but both left and right half of mandibles were equal in N2 (Table 1). 

Two separated alimentary tracts were seen in a common abdomen with only one spleen. There were two unequal livers holding one gall bladder. A uterus with its ovaries and also one urinary bladder were seen in N1. The bladder received four ureters from four kidneys. One diaphragm separated common thorax and abdominal cavities showing only one aortic hiatus but two esophageal hiatus, and two caval foramina. 

**Fig. 5:**
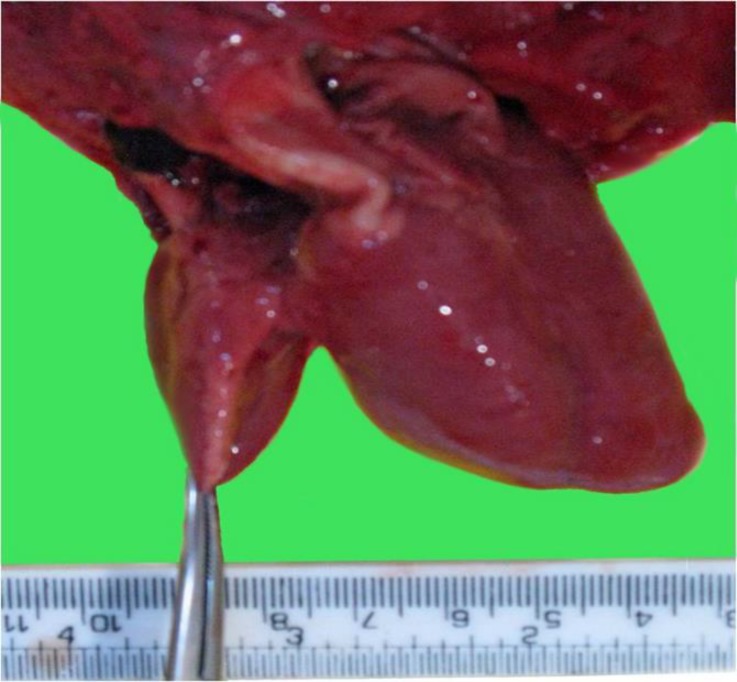
The photograph showing two hearts of unequal sizes after removal of the pericardium

Two unequal hearts locating in common pericardium and two paired lungs were observed. They were conjoined at their bases. Thus, the common atria of smaller heart conjoined with the right part of the larger heart (Figs. 5). 

The smaller heart belonged to N2, and had a common atrium and ventricle. One pulmonary trunk was derived from the common ventricle of the smaller heart which had an extra common carotid branch. The larger heart had normal cardiac vessels and more over two subclavian branches for N2. 

## Discussion

The fusion of two new born lambs was severe extending from the cranial region of the thorax to the caudal region of the pelvic (thoraco-omphalopygopagus). The result of this fusion created a common thorax and a common abdomen. Cywes* et al.* noted that the greater extent of the thoracic cage fusion, the more chance of associated severe abnormality.^7^

Two major abnormalities are noticeable in this study. The first one is the lack of one genital system and bladder; this in N2 may be due to the lack of umbilical cord. The second one is concerned with great arteries abnormalities.

Cywes* et al.* reported that in thoracopagus twins, the hearts were of paramount importance as conjunction was usually fatal being associated with major congenital defects.^7^ Ambar* et al.* also pointed out that cardiac abnormalities were present in any type of fusion. ^10^ Thoracopagus twins have the incidence of cardiac anomalies with a 90% incidence of shared pericardium. 

Two unequal hearts in size with unusual branching of greater arteries and connection between them leads to mix the oxygenic and non-oxygenic blood that might have caused the death of the lambs. This is the first report of such monster lambs in Iran.
